# Changes in the membrane lipid composition of a *Sulfurimonas* species depend on the electron acceptor used for sulfur oxidation

**DOI:** 10.1038/s43705-022-00207-3

**Published:** 2022-12-24

**Authors:** Su Ding, Jan V. Henkel, Ellen C. Hopmans, Nicole J. Bale, Michel Koenen, Laura Villanueva, Jaap S. Sinninghe Damsté

**Affiliations:** 1 NIOZ Royal Institute for Sea Research, Department of Marine Microbiology and Biogeochemistry, Texel, The Netherlands; 2 Department of Bioscience, Center for Geomicrobiology, Aarhus University, Aarhus, Denmark; 3 Biological Oceanography, Leibniz Institute for Baltic Sea Research Warnemünde, Rostock, Germany; 4 Department of Earth Sciences, Faculty of Geosciences, Utrecht University, Utrecht, The Netherlands

**Keywords:** Biogeochemistry, Biogeochemistry, Biogeochemistry, Water microbiology, Marine microbiology

## Abstract

*Sulfurimonas* species are among the most abundant sulfur-oxidizing bacteria in the marine environment. They are capable of using different electron acceptors, this metabolic flexibility is favorable for their niche adaptation in redoxclines. When oxygen is depleted, most *Sulfurimonas* spp. (e.g., *Sulfurimonas gotlandica*) use nitrate (${{{{{{{\mathrm{NO}}}}}}}}_3^ -$) as an electron acceptor to oxidize sulfur, including sulfide (HS^-^), S^0^ and thiosulfate, for energy production. *Candidatus* Sulfurimonas marisnigri SoZ1 and *Candidatus* Sulfurimonas baltica GD2, recently isolated from the redoxclines of the Black Sea and Baltic Sea respectively, have been shown to use manganese dioxide (MnO_2_) rather than ${{{{{{{\mathrm{NO}}}}}}}}_3^ -$ for sulfur oxidation. The use of different electron acceptors is also dependent on differences in the electron transport chains embedded in the cellular membrane, therefore changes in the membrane, including its lipid composition, are expected but are so far unexplored. Here, we used untargeted lipidomic analysis to reveal changes in the composition of the lipidomes of three representative *Sulfurimonas* species grown using either ${{{{{{{\mathrm{NO}}}}}}}}_3^ -$ and MnO_2_. We found that all *Sulfurimonas* spp. produce a series of novel phosphatidyldiazoalkyl-diacylglycerol lipids. *Ca*. Sulfurimonas baltica GD2 adapts its membrane lipid composition depending on the electron acceptors it utilizes for growth and survival. When carrying out MnO_2_-dependent sulfur oxidation, the novel phosphatidyldiazoalkyl-diacylglycerol headgroup comprises shorter alkyl moieties than when sulfur oxidation is ${{{{{{{\mathrm{NO}}}}}}}}_3^ -$-dependent. This is the first report of membrane lipid adaptation when an organism is grown with different electron acceptors. We suggest novel diazoalkyl lipids have the potential to be used as a biomarker for different conditions in redox-stratified systems.

## Introduction

Chemoautotrophic bacteria of the genus *Sulfurimonas* [class Campylobacterota, according to the NCBI classification, [1, 2] play an important role in sulfur oxidation in sulfidic habitats, such as stratified marine waters, anoxic sediments, and hydrothermal deep-sea vents, as well as in some terrestrial environments [3]. For example, along the Namibian shelf, Lavik, et al. [4] reported that occasionally occurring, large-scale sulfidic water masses were detoxified by oxidation of sulfide (HS^-^) using nitrate $({{{{{{{\mathrm{NO}}}}}}}}_3^ - )$ by two groups of bacteria: *Sulfurimonas* spp. and bacteria falling in the gammaproteobacterial sulfur oxidizer cluster. Within and below the pelagic redoxcline of the Baltic Sea, where a stable redoxcline separates deep anoxic sulfidic water from oxygenated surface water [5], *Sulfurimonas* spp. accounted for up to 15% of total prokaryotic abundance. Here, Grote, et al. [6] isolated *S. gotlandica* GD1^T^, a denitrifying chemolithotrophic sulfide oxidizer. In oxygen deficient systems, sulfur cycling has been shown to be tightly linked to the nitrogen cycle without affecting chemical gradients, so called cryptic cycling [e.g., [7, 8].

In the stratified water columns of both the Baltic Sea and the Black Sea, ${{{{{{{\mathrm{NO}}}}}}}}_3^ -$ and nitrite (${{{{{{{\mathrm{NO}}}}}}}}_2^ -$) often disappear before the first appearance of HS^-^ [9, 10]. Geochemical water column profiles suggest that extensive cycling of dissolved and particulate manganese (Mn) may quantitatively account for the oxidation of HS^-^, thereby shuttling oxidative potential of O_2_ and ${{{{{{{\mathrm{NO}}}}}}}}_3^ -$ over several meters distance. This mechanism, as well as a potential biological catalyzation, was proposed earlier because chemoautotrophic HS^-^ oxidation could not be linked to O_2_ or ${{{{{{{\mathrm{NO}}}}}}}}_3^ -$ [10–12]. Recently, Henkel, et al. [13] isolated *Ca*. Sulfurimonas marisnigri SoZ1 from the redoxcline of the central Black Sea. This bacterium can couple the oxidation of reduced sulfur compounds, including HS^-^, S^0^ and thiosulfate, to the reduction of MnO_2_. The biologically catalyzed oxidation of HS^-^ by *Ca*. S. marisnigri SoZ1 is faster than the abiotic oxidation of HS^-^ with MnO_2_ [14] and yields ${{{{{{{\mathrm{SO}}}}}}}}_4^{2 - }$ as the prominent end product, while elemental sulfur (S^0^) accumulates in the abiotic reaction [13, 15, 16]. Combining the cellular abundance of *Sulfurimonas* spp. in the Black Sea redoxcline with rates of MnO_2_ dependent HS^-^ oxidation by *Ca*. S. marisnigri SoZ1 in a reaction diffusion model yielded adequate rates to counterbalance the HS^-^ flux, resulting in the measured HS^-^ concentration profile, in contrast to a pure abiotic reaction [14]. Hence, this process is likely to play a prominent role in HS^-^ oxidation at the chemocline of the Black Sea. Likewise, *Ca*. Sulfurimonas baltica GD2 was isolated from the redoxcline of the Gotland Deep in the Baltic Sea [17]. These studies revealed the important role of MnO_2_-dependent *Sulfurimonas* spp. in biogeochemical cycling in redox-stratified systems [14]. Changes in the electron acceptor are associated with differences in the electron transport chain (ETC), which is embedded in the cellular membrane. Protein complexes of the ETC are expected to interact with and influence the lipid composition of the membrane, which is partially responsible for the fluidity of the membrane [18]. Nevertheless, few studies have addressed potential changes in the membrane lipid composition upon changes in the electron acceptor as a response to maintain the cell homeostasis.

Structurally diverse microbial lipids play important roles as the building blocks of membranes and play a role in energy storage, signaling and modulation of protein activity [19]. Many microorganisms maintain their membrane functionality, permeability, and fluidity during changing environmental conditions by membrane adaptation [20, 21] through the regulation of its lipid composition [e.g., [22–25]. For instance, when phosphorus is limited, phytoplankton and some bacteria can use non-phosphorus lipids to replace phospholipids for survival [23, 26, 27]. However, the membrane lipid composition of *Sulfurimonas* spp., and potential differences when using different electron acceptors to oxidize HS^-^, are unknown. Until now, only a few studies have reported the predominant cellular fatty acids of *Sulfurimonas* spp. [e.g., [28–30], but their intact polar lipid composition, including both the fatty acid side chain and their linked polar headgroups, remains to be studied. Because of their relatively high abundance in redox-stratified waters, *Sulfurimonas* spp. are likely to contribute substantially to the lipidome of these environments. Recent advances in the field of lipidomics, using non-targeted approaches combined with computational methods, allows for comprehensive lipidome profiling [31–34].

Here, we examined the lipidome of three *Sulfurimonas* species, *S. gotlandica* GD1^T^ and *Ca. S*. baltica GD2, both isolated from water column of the Baltic Sea, and *Ca. S*. marisnigri SoZ1 isolated from the water column of the Black Sea, cultured with either ${{{{{{{\mathrm{NO}}}}}}}}_3^ -$ or MnO_2_ as electron acceptor. Our aim was to investigate how *Sulfurimonas* spp. adjust their membrane lipid composition when using different electron acceptors (${{{{{{{\mathrm{NO}}}}}}}}_3^ -$ and MnO_2_), in order to determine potential microbial adaptations of the membrane linked to different metabolisms.

## Methods and materials

### Cultures

Anoxic medium was prepared with a salinity of 21, 14 and 10 psu for *Ca*. S. marisnigri SoZ1, *Ca*. S. baltica GD2 and *S. gotlandica* GD1^T^ respectively as described before [17] with minor changes. We used 2 L glass bottles closed with butyl rubber stoppers for both MnO_2_ and ${{{{{{{\mathrm{NO}}}}}}}}_3^ -$ conditions with Ca. S. marisnigri SoZ1 and Ca. S. baltica GD2. S. gotlandica was cultivated in 100 mL serum bottles closed with butyl rubber stoppers. We raised the concentration of NH_4_Cl and Na_2_HPO_4_ from 0.02 mM and 0.01 mM to 2.5 mM and 0.2 mM, respectively. These adjustments were made to exclude deficiencies of macro nutrients during growth and should therefore reduce differences in lipid composition of cultures. Cultures were obtained from the internal culture collection of the IOW, which are also available at the German Collection of Microorganisms and Cell Cultures GmbH (DSMZ) and at the Japan Collection of Microorganisms (JCM) with the identifiers JCM 39139 and DSM 111879 (*Ca*. S. marisnigri SoZ1), JCM 39138 and DSM 111898 (*Ca*. S. baltica GD2) and JCM16533 and DSM 19862 (*S. gotlandica* GD1^T^). *Ca*. S. marisnigri SoZ1 and *Ca*. S. baltica GD2 were cultured with either 3 mM thiosulfate (${{{{{{{\mathrm{S}}}}}}}}_2{{{{{{{\mathrm{O}}}}}}}}_3^{2 - }$) and 5 mM MnO_2_ (Mn-reducing conditions) or 5 mM ${{{{{{{\mathrm{S}}}}}}}}_2{{{{{{{\mathrm{O}}}}}}}}_3^{2 - }$ and 10 mM ${{{{{{{\mathrm{NO}}}}}}}}_3^ -$ (${{{{{{{\mathrm{NO}}}}}}}}_3^ -$ reducing conditions). *S. gotlandica* GD1^T^ was amended with 15 mM ${{{{{{{\mathrm{NO}}}}}}}}_3^ -$ and 10 mM ${{{{{{{\mathrm{S}}}}}}}}_2{{{{{{{\mathrm{O}}}}}}}}_3^{2 - }$. *S. gotlandica* GD1 grows faster and has a substantially higher growth efficiency with nitrate than Ca. S. marisnigri SoZ1 and Ca. S. baltica GD2. This is because *S. gotlandica* GD1 is a complete denitrifier (${{{{{{{\mathrm{NO}}}}}}}}_3^ -$ to N_2_), while Ca. S. marisnigri SoZ1 and Ca S. baltica GD2 can only reduce ${{{{{{{\mathrm{NO}}}}}}}}_3^ -$ to ${{{{{{{\mathrm{NO}}}}}}}}_2^ -$ [17]. Since *S. gotlandica* grows faster than the other strains, the initial ${{{{{{{\mathrm{NO}}}}}}}}_3^ -$ and S_2_O_3_^2-^ concentrations were higher to prolong the exponential growth phase. Concentrations of ${{{{{{{\mathrm{NO}}}}}}}}_3^ -$ (and therefore also S_2_O_3_^2-^) in cultivations of Ca. S. marisnigri SoZ1 and Ca. S. baltica GD2 are lower to exclude a putative toxification by ${{{{{{{\mathrm{NO}}}}}}}}_2^ -$. Cell numbers were estimated after 8 days of growth and the biomass were harvested at day 12 at 10 °C in the dark. Cellular abundance of *Ca*. S. marisnigri SoZ1 and *Ca*. S. baltica GD2 were ~5 × 10^6^ cells mL^−1^ under Mn-reducing conditions and ~5 × 10^5^ cells mL^−1^ under ${{{{{{{\mathrm{NO}}}}}}}}_3^ -$ reducing conditions (*Ca*. S. baltica GD2 only). Very few cells were visible under ${{{{{{{\mathrm{NO}}}}}}}}_3^ -$ reducing conditions with *Ca*. S. marisnigri SoZ1. *S. gotlandica* GD1^T^ reached a cellular abundance of ~4 × 10^7^ cells mL^−1^ at day 8. Incubation was continued until day 10 at which the black color of Mn-containing incubations became slightly brownish-gray, indicating the precipitation of Ca-rich Mn-carbonates at the end of the exponential growth phase [13, 17]. Cells were harvested in 50 mL centrifugation tubes at 4060 x g for 15 minutes at 10 °C, pooled and stored at −20 °C before freeze drying and shipping to the NIOZ for lipidome analysis.

### Lipidome analysis

The cells of three representative *Sulfurimonas* species (*Ca. S*. marisnigri SoZ1, *Ca. S*. baltica GD2 and *S. gotlandica* GD1^T^) were collected and freeze dried for lipidome analysis. A detailed description of sample extraction and ultra-high performance liquid chromatography coupled to high-resolution tandem mass spectrometry (UHPLC-HRMS^2^) analysis and initial data processing is given in Bale, et al. [35]. Briefly, freeze-dried samples were extracted using a modified Bligh-Dyer procedure. They were extracted ultrasonically for 10 min, twice in a mixture of methanol, dichloromethane and phosphate buffer (2:1:0.8, v-v:v) and twice with a mixture of methanol, dichloromethane and aqueous trichloroacetic acid solution (TCA) pH 3 (2:1:0.8, v-v:v). The organic phase was separated by adding additional dichloromethane and buffer to a final solvent ratio of 1:1:0.9 (v:v) and were re-extracted three times with dichloromethane and dried under a stream of N_2_ gas. The extract was redissolved in a mixture of MeOH:DCM (9:1, v-v) and were filtered through 0.45 *µ*m regenerated cellulose syringe filters (4 mm diameter; Grace Alltech). The extracts were then analyzed using Agilent 1290 Infinity I UHPLC coupled to a Q Exactive Orbitrap MS (Thermo Fisher Scientific, Waltham, MA). The output data files generated by the UHPLC-HRMS^2^ analyses were further processed using MZmine software [36]. Process steps included mass peak detection, chromatogram building and deconvolution, isotope grouping, ion component alignment and gap filling [34]. The relative abundance of components was obtained after processing and the combined dataset of MS/MS spectra were analyzed using the Feature Based Molecular Networking tool [33] through the Global Natural Product Social Molecular Networking (GNPS) platform [34] to build molecular networks of the detected components in the dataset (https://gnps.ucsd.edu/ProteoSAFe/status.jsp?task=f775f785684e4cc1b9e133d7abcc6f00). Details can be found in Ding, et al. [37]. It should be noted that many of the lipids detected in this study have not been described previously and hence authentic standards for absolute quantification are not available. Therefore, the lipid compositions were examined in terms of their peak area response. Thus, the relative peak area does not necessarily reflect the actual relative abundance of the different lipids, however, this method allows for some comparison between the samples analyzed in this study. Due to the extraction and analytical methods, and based on annotation from Ding, et al. [37], most of the ion components from the molecular network we generated were lipids and contaminants, thus we used the term “lipidome” for parts of the results and discussion where the lipids are discussed.

## Results and discussion

### Lipidome of three representative *Sulfurimonas* species

Three representative *Sulfurimonas* species were cultured with thiosulfate using ${{{{{{{\mathrm{NO}}}}}}}}_3^ -$ or MnO_2_ as electron acceptors. Thiosulfate was used instead of HS^- ^as it is a more convenient electron donor, being non-toxic even in higher concentrations, and non-reactive with the MnO_2_ [13]. Cells were harvested after 12 days of growth, which corresponds to the late exponential or early stationary phase [17]. The growth stages of *Ca*. S. marisnigri SoZ1 and *Ca*. S. baltica GD2 were comparable due to the reliable change in color of the growth media in the late exponential growth phase, and the fact that the time needed to reach final cell densities with ${{{{{{{\mathrm{NO}}}}}}}}_3^ -$ does not differ from that with MnO_2_ based on our experience with these strains. Similar to the previous studies, growth yields of *Ca*. S. marisnigri SoZ1 and *Ca*. S. baltica GD2 cultured with ${{{{{{{\mathrm{NO}}}}}}}}_3^ -$were substantially lower compared to cultivation with MnO_2_ as terminal electron acceptor [17]. The biomass yield of *Ca*. S. marisnigri SoZ1 grown with ${{{{{{{\mathrm{NO}}}}}}}}_3^ -$ was too low for a precise lipidomic analysis, therefore it is excluded for the following analysis. *S. gotlandica* GD1^T^ is unable to grow with MnO_2_ [17]. Since only *Ca*. S. baltica GD2 could be cultured with both ${{{{{{{\mathrm{NO}}}}}}}}_3^ -$ and MnO_2_ conditions, this is the only species that could be examined in terms of its membrane adaptation to a change in electron acceptor. The results of *Ca*. S. marisnigi SoZ1 grown with MnO_2_ and *S*. gotlandica GD1 grown with ${{{{{{{\mathrm{NO}}}}}}}}_3^ -$ provide complementary information about their lipid specificities and are thus interesting for comparison.

Recently, we established a workflow that provides a substantially expanded view of the molecular composition of the microbial lipidome in environmental settings [37]. Here we used the same workflow for our culture lipidome analysis. The complete dataset produced by analysis with high performance liquid chromatography coupled to high resolution tandem mass spectrometry (HPLC–HRMS^2^) of the Bligh-Dyer lipid extracts of the cultures contained 9936 unique ion components, of which 4282 ion components (43%) occurred in structure-similarity groupings in the molecular network, while 5654 ion components occurred as singletons (i.e., components without molecular relatives). A search in the Global Natural Products Social Molecular Networking (GNPS) library [34] resulted in only 167 spectral annotations (<2%). Other than ca. 20 contaminants (e.g., plasticizers), the majority of these annotations were those of well-known glycerol-based lipids: phosphatidylethanolamines (PE-DAGs), phosphatidyl glycerols (PG-DAGs) and triayclglycerols (TAGs). However, the vast majority of ion components was left unannotated. Lipidome annotation remains a bottleneck in marine microbiology lipidomic studies because public databases in this field are poorly populated [35, 37].

**Fig. 1:**
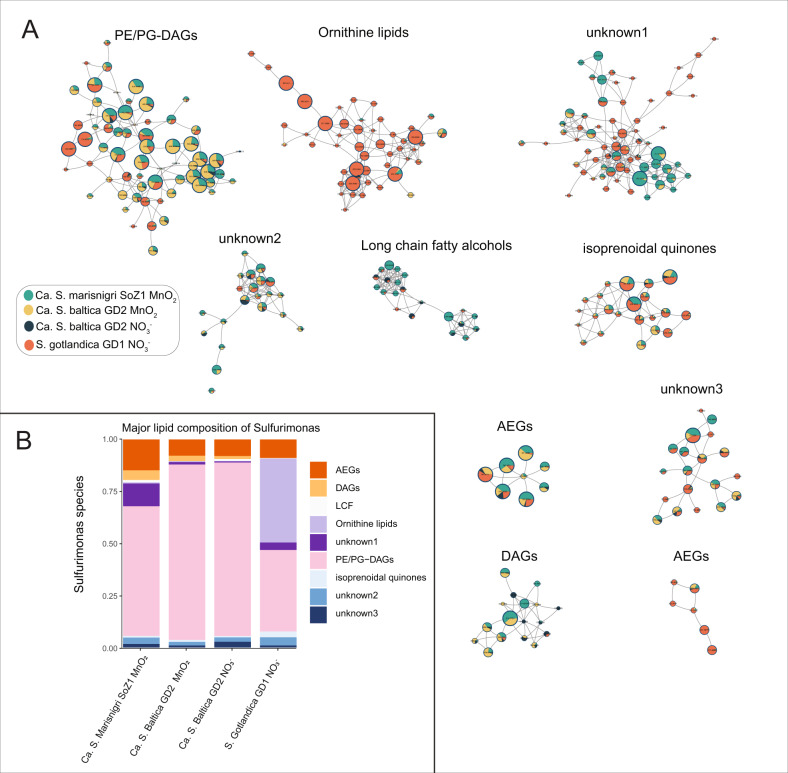
**A** Molecular network of the major lipid classes in the *Sulfurimonas* species (*Ca. S*. marisnigri SoZ1, *Ca. S*. baltica GD2 and *S. gotlandica* GD1^T^) using ${{{{{{{\mathrm{NO}}}}}}}}_3^ -$ or MnO_2_ as electron acceptors. Nodes represent MS/MS spectra of ion components (lipids), which are connected based on spectral similarity (cosine > 0.6). Nodes are filled with pie charts which contain four different colors representing the fractional abundance of the lipid (based on peak intensity) among three *Sulfurimonas* species with two different treatments. The size of each pie charts represents for the summed intensity of the ion component in all the species using various electron acceptors. The aim of this figure is not to compare the abundance of lipids among different *Sulfurimonas* species, rather than that it provides information on whether specific *Sulfurimonas* spp. under certain conditions have the ability to synthesize specific lipids. The spatial orientation of the nodes in the MS/MS network is randomly generated by Cytoscape [58, 59] and does not relate to relationships between the subnetworks. Lipid classes (clusters) with annotation are either tentatively identified in this study or have been annotated from the GNPS library (see the method for details). **B** Major lipid class composition (based on peak intensity). DAG diacylglycerol, AEG acyletherglycerol, LCF long chain fatty acids, PE phosphatidylethanolamine, PG phosphatidylglycerol. There are also three unknown lipid classes, the structure of unknown lipid 1 will be discussed in Fig. 3. The other two lipid classes are lipids with polar headgroups that we could not identify based on their mass spectra. However, their mass spectra indicate that they comprise a DAG core (Fig. S1).

The major lipid classes detected in the *Sulfurimonas* spp. were PE/PG-DAGs, diacylglycerols (DAGs) and acyl ether glycerols (AEGs) without a polar head group, ornithine lipids, isoprenoidal quinones, long-chain fatty acids and some unknown lipids (Fig. 1A). The distribution of major lipid classes, based on the peak intensity, were similar between Ca. S. baltica GD2, grown with ${{{{{{{\mathrm{NO}}}}}}}}_3^ -$ and with MnO_2_ (Fig. 1B). PE/PG-DAGs were dominant among all the lipids, accounting for >80% of total lipids. AEGs were the second most abundant lipid class, accounting for nearly 8% of total lipids. Isoprenoid quinones and some unknown lipids accounted for 0.5–3% of total lipids. PE/PG-DAGs were also the most abundant group of lipids in *Ca*. S. marisnigi SoZ1 grown with MnO_2_, comprising 62% of the total lipids, followed by 15% AEGs and 11% unknown lipids. In contrast to Ca. S. baltica GD2 and *Ca*. S. marisnigi SoZ1, PE/PG-DAGs comprised only 39% of total lipids in *S. gotlandica* GD1^T^ grown with ${{{{{{{\mathrm{NO}}}}}}}}_3^ -$. Ornithine lipids were almost exclusively produced by *S. gotlandica* GD1^T^ grown with ${{{{{{{\mathrm{NO}}}}}}}}_3^ -$ (Fig. S2), accounting for 40% of the total lipids. Ornithine lipids are phosphorus-free amino intact polar lipids. which are relatively common in bacteria, but are absent in eukaryotes and archaea [38]. About 50% of sequenced bacteria are suggested to be able to synthesize ornithine lipids under certain growth conditions [24, 39].

The lipidome differences between Ca. S. marisnigri SoZ1 and S. gotlandica GD1 may be attributed to membrane differences between the species or to factors such as differences in the growth media. Differences in the lipidome of Ca. S. baltica GD2, depending on whether they were grown with ${{{{{{{\mathrm{NO}}}}}}}}_3^ -$ or MnO_2_, are not evident from the total lipid distribution, so we subsequently examined the compositional differences within specific lipid classes.

**Fig. 2:**
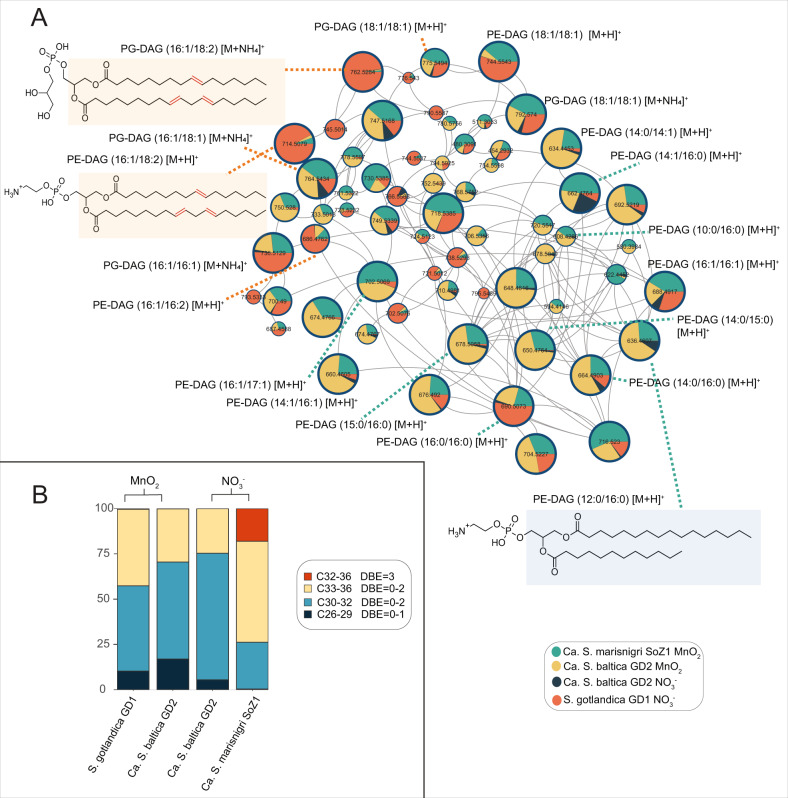
**A** Molecular subnetwork of PE-DAGs and PG-DAGs. Pie charts shown in the subnetwork are the same as nodes shown in Fig. 1, representing fractional abundance of different individual lipids. The size of the pie charts represents the summed intensity of the ion component in all the species grown with various electron acceptors. Colors of the pie chart represent the fractional abundance of this ion component among all the *Sulfurimonas* species and different treatment. Numbers in the pie charts are the precursor mass of the lipids. Double bonds of the lipid structure are localized tentatively to show their numbers rather than their exact position.; **B** PE-DAG and PG-DAG lipids and their fractional abundance (of total PE-DAGs and PG-DAGs in %) shown in subnetwork of Fig. 2A. The numbers in the labels stand for the total number of carbon atoms of the two acyl chains (C26–36) and their summed double bonds (DBE = 0–3).

PE-DAGs and PG-DAGs are the major components of most bacterial membranes [40, 41] and this also holds for the investigated *Sulfurimonas* species (Fig. 2B). A subnetwork of diverse PE- and PG-DAGs (Fig. 2A) was shown in the molecular network. The acyl moieties (i.e., derived from the esterified fatty acids) of these abundant PEs and PGs had 0–3 double bond equivalents (DBEs) and contained 26–36 carbon atoms (i.e., the sum of the two acyl chains). The majority (i.e., 50–70%) of the PE- and PG-DAGs had 30–32 total acyl carbons with 0 to 2 DBEs. The most dominant individual PE- and PG-associated fatty acids were C_14_, C_15_ and C_16,_ saturated and monounsaturated fatty acids, in agreement with a previous study that reported that the dominant fatty acid of *S. gotlandica* GD1^T^ was C_16:1_ (66% of total), with smaller amounts of C_18:1_ and C_16:0_ fatty acids [30]. Our detailed analyses revealed a minority of smaller PE- and PG-DAGs, containing 26–29 total acyl carbons and with 0–1 DBEs. In the only *Sulfurimonas* species that was cultivatable under both ${{{{{{{\mathrm{NO}}}}}}}}_3^ -$ and MnO_2_ conditions, i.e., *Ca. S*. baltica GD2, the proportion of these smaller PE- and PG-DAGs (26–29 acyl carbons, 0–1 DBEs) was 5.5% of total PE/PG-DAGs under ${{{{{{{\mathrm{NO}}}}}}}}_3^ -$ conditions and 17.0% under MnO_2_ conditions, suggesting a phospholipid acyl chain adaption in *Ca. S*. baltica GD2 depending on the electron acceptor used.

### Novel lipids found in the *Sulfurimonas* species

**Fig. 3:**
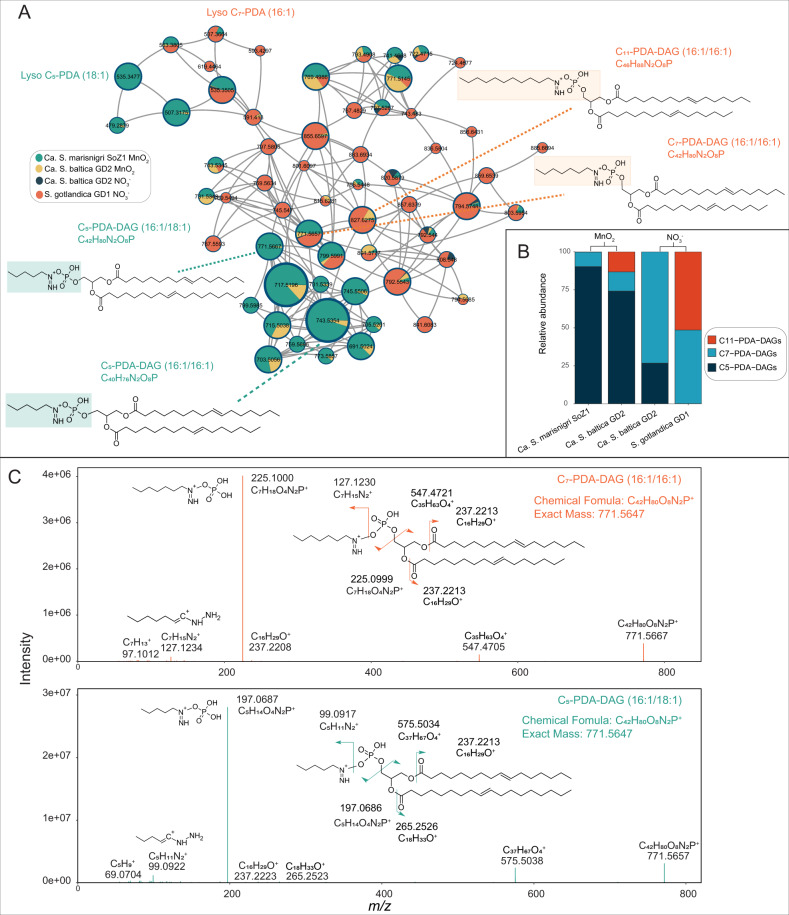
**A** The molecular subnetwork of the tentatively identified PDA-DAG lipids. Pie charts shown in subnetwork are the same as nodes shown in Fig. 1, representing different lipid structures. The size of the pie charts represents the summed intensity of all the species using various electron acceptors. Colors of pie chart represents the fractional abundance of this lipid among all the *Sulfurimonas* species grown at various conditions. **B** PDA-DAG lipid structures and their fractional abundance of total PDA-DAGs found in subnetwork Fig. 3. **C** MS^2^ spectra of two PDA-DAGs that have the same elemental composition (EC of C_42_H_80_O_8_N_2_P^+^; *m/z* 771.5647) but differ in the size of the alkyl chains of the polar headgroup and the number of carbon atoms of the acyl moieties.

A subnetwork (>60 components; Fig. 3A, B) entirely populated by unknown lipids (labeled “unknown1” in Fig. 1) revealed a clear division between *Ca*. S. baltica GD2 grown with either ${{{{{{{\mathrm{NO}}}}}}}}_3^ -$ or with MnO_2_ as the electron acceptor. The composition of this unknown lipid network was also noticeably different between *Ca*. S. marisnigi SoZ1 grown with MnO_2_ and *S*. gotlandica GD1 grown with ${{{{{{{\mathrm{NO}}}}}}}}_3^ -$. One of the central and relatively abundant nodes, with a lipid structure occurring in all *Sulfurimonas* spp. grown with ${{{{{{{\mathrm{NO}}}}}}}}_3^ -$ or with MnO_2_, represents a compound with a molecular ion at *m/z* 771.5657 (as per the molecular network, cf. Fig. 3) eluting at 17.0–17.5 min (Fig. S3). This component was assigned an elemental composition (EC) of C_42_H_80_O_8_N_2_P^+^ (Δmmu = 1.00; the difference in millimass; [calculated mass – observed mass] x 1000). Upon MS^2^ fragmentation (Fig. 3C) a dominant fragment ion at *m/z* 225.1000 with an EC of C_7_H_18_O_4_N_2_P^+^ (Δmmu = 0.13) was formed, likely representing the polar headgroup, and a relatively minor and complimentary fragment at *m/z* 547.4704 (EC C_35_H_63_O_4_^+^; Δmmu = −1.59) representing the diacyl glycerol core containing the esterified fatty acids with a combined total number of carbon atoms of 32 and 2 DBEs. A single fragment ion at *m/z* 237.2208 with EC of C_16_H_29_O^+^ suggests the diacyl glycerol is composed of two C_16:1_ fatty acids. An apparent loss of H_3_PO_4_ from the main headgroup ion at *m/z* 225.1000 results in a fragment ion at *m/z* 127.1234, (EC C_7_H_15_N_2_^+^; Δmmu = 0.42) and a further loss of N_2_H_2_, suggesting the two nitrogen are adjacent to each other in the molecule, results in a fragment ion at *m/z* 97.1012 with an EC of ${{{{{{{\mathrm{C}}}}}}}}_7{{{{{{{\mathrm{H}}}}}}}}_{13}^ +$ (Δmmu = −0.52). We, therefore, tentatively identify this lipid as a C_16:1_/C_16:1_ phosphatidyldiacylglycerol bound to a diazoheptane moiety. The dominance of the fragment ion representing the headgroup in the MS^2^ spectrum is very similar to the MS^2^ behavior of polar headgroups with a quaternary ammonium (*i.e*., phosphocholine headgroups) and we, therefore, suggest the diazoheptane moiety is bound not via the terminal N, but to the adjacent N resulting in a quaternary ion. The extracted ion chromatogram of *m/z* 771.5667 (as per the molecular network, cf. Fig. 3) revealed several additional peaks at 18.2–18.5 min (Fig. S3) in the *Sulfurimonas* species grown with MnO_2_ with identical *m/z* and assigned EC. Interestingly, their fragmentation mass spectrum showed a similar pattern as described for the peak at 17.0–17.5 min (Fig. 3C), however the fragment ion representing the headgroup was now observed at *m/z* 197.0687 (Δmmu = −0.39) and the fragment ion related to the carbon chain in the hydrazine moiety was observed at *m/z* 69 with an EC of ${{{{{{{\mathrm{C}}}}}}}}_5{{{{{{{\mathrm{H}}}}}}}}_9^ +$. The fragment ion representing the diacyl core was now observed at *m/z* 575.5038 (C_37_H_67_O_4_^+^). In addition to a fragment indicating a C_16:1_ fatty acid, an additional fragment was observed at *m/z* 265 representing a C_18:1_ fatty acid. We therefore assigned this lipid as a phosphatidyldiazopentane diacylglycerol (16:1/18:1). Further inspection of the subnetwork revealed a series of diazoalkyl lipids varying both in the carbon number of the alkylhydrazine headgroup moiety from 5 to 11 and in their acyl carbon atoms from 34 to 36, leading to their tentative assignment as phosphatidyldiazoalkyl-diacylglycerols (hereafter, PDA-DAGs). The structural identification here is tentative, further structural elucidation methods (e.g., nuclear magnetic resonance [NMR] spectroscopy) would be required to confirm the proposed structures and elucidate the position of the various functional groups.

The PDA-DAGs with a short C5 alkyl chain in the headgroup were dominant in the two cultures that used MnO_2_ as the electron acceptor: Ca. S. marisnigri (90% of total PDA-DAGs) and Ca. S. baltica (74% of total PDA-DAGs) (Fig. 3B). In contrast, Ca. S. baltica GD2, grown with ${{{{{{{\mathrm{NO}}}}}}}}_3^ -$, produced much less C_5_ PDA-DAGs (27% of total PDA-DAGs), and mostly C_7_ PDA-DAGs, (73%) suggesting a membrane lipid adaptation depending on electron acceptors of this *Sulfurimonas* species. S. gotlandica GD1, unable to grow with MnO_2_ as electron acceptor, produced predominantly PDA-DAGs with a headgroup containing a longer alkyl chain (C7 and C11; 49% and 51% of total PDA-DAGs, respectively), while C_5_ PDA-DAGs were almost absent (0.1%). The PDA-DAGs composition of Ca. S. marisnigi SoZ1 grown with MnO_2_ and on S. gotlandica GD1 grown with ${{{{{{{\mathrm{NO}}}}}}}}_3^ -$ were potentially attributed to their species specificities.

A search using the Mass Spectrometry Search Tool (MASST) of over 1000 public LC-MS datasets available in the GNPS platform database (including lipidomes, metabolomes etc.) was performed to see whether lipids structurally similar to the PDA-DAGs detected in this study of *Sulfurimonas* cultures had been detected in other organisms or environments [34, 37]. However, none of the databases showed matches to these unique lipids, suggesting that either it was due to a database bias or these novel components are potentially indicative of this taxonomic group, because of their specific metabolism or the environmental adaptation to their niche (redoxcline). Indeed, natural products containing a diazo group are rare [42–44] and their presence in the headgroups of microbial membrane lipids has not been reported before to the best of our knowledge. Although their structure remains tentative, one could speculate about their potential biosynthesis. There are enzymes reported that convert an amine group into a diazo functional group [42]. Primary alkyl amines can be enzymatically produced from fatty acids [45] or by decarboxylation of aliphatic amino acids [46]. The only truly enigmatic biochemical reaction would be the coupling of the diazo functional group with the phosphate moiety of the intact polar lipids.

### Potential causes for membrane lipid adaptation with different electron acceptors

Our results revealed marked changes in the composition of (i) the short alkyl chain of the headgroup of PDA-DAG lipids and (ii) the acyl moieties of the PE- and PG-DAGs of *Ca*. S. baltica GD2 depending on the available terminal electron acceptor. It is well established from culture studies that temperature, pressure, pH, growth rate and nutrients are key factors in regulating the fatty acid and headgroup composition of membrane lipids of microorganisms [47–50]. In order to maintain membrane fluidity and permeability at the cultured conditions where pressure is elevated and temperature is low, microorganisms modify their fatty acid composition by increasing the amount of unsaturation and decreasing the chain lengths [21, 51, 52]. Such phenomena were also observed in the natural environment such as the ocean and global soils [53–55]. However, in studies of the eastern subtropical South Pacific, it has been shown that the chain length and degree of unsaturation can be an inherent biochemical property of a lipid class, rather than a function of temperature or pressure [56]. The sequential utilization of terminal electron acceptors is a function of potential energy yield based on thermodynamics which results in the mostly vertically progressive depletion of electron acceptors from O_2_ to ${{{{{{{\mathrm{NO}}}}}}}}_3^ -$, Mn(IV), Fe(III) and ${{{{{{{\mathrm{SO}}}}}}}}_4^{2 - }$ [57]. Our results suggest that *Ca*. S. baltica GD2 produces shorter chain length lipids when using MnO_2_ instead of ${{{{{{{\mathrm{NO}}}}}}}}_3^ -$ for energy production. This may indicate that *Ca*. S. baltica GD2 modify their membrane permeability by changing their lipid composition to achieve electron exchange under MnO_2_ conditions or due to the different membrane proteins associated with lipids mediating these reactions. To our knowledge, this is the first report of changes in the microbial membrane lipid composition in response to different electron acceptors. Further studies are required to screen for the presence of PDA-DAGs in the natural environment and to determine the biological mechanisms underlying their presence in *Sulfurimonas*.

## Conclusion

This study showed that the membrane lipid composition of a *Sulfurimonas* species*, Ca*. S. baltica GD2, depends on the electron acceptor used during culturing, either ${{{{{{{\mathrm{NO}}}}}}}}_3^ -$ or MnO_2_. Differences in the lipid composition of other two species, *Ca*. S. marisnigi SoZ1 grown with MnO_2_ and on *S*. gotlandica GD1 grown with ${{{{{{{\mathrm{NO}}}}}}}}_3^ -$ were likely attributed to their species specificities. A range of novel diazoalkyl phospholipids were tentatively identified by using molecular network and high-resolution mass spectrometry. We observed that when carrying out MnO_2_-dependent sulfur oxidation, *Ca*. S. baltica GD2 possesses shorter acyl (fatty acid) chain lengths in its PE- and PG-DAGs and shorter alkyl chains in its phosphatidyldiazoalkyl-diacylglycerols lipid polar headgroups, compared to the ones produced when grown with ${{{{{{{\mathrm{NO}}}}}}}}_3^ -$. These chain length modifications may be utilized by the cells to maintain membrane homeostasis when using MnO_2_ instead of ${{{{{{{\mathrm{NO}}}}}}}}_3^ -$ for energy production. Advances in untargeted lipidomic approaches and the development of computational tools will enable the more-efficient characterization of microbial lipidome and the search for unknowns, leading to a better understanding of their functions and metabolism under different environmental conditions.

## Supplementary information

Supplementary Information

Supplementary Tables

## Data Availability

The HPLC-MS/MS datasets generated during the current study are available from the corresponding author on reasonable request.
