# Supplementary material for: Changes in the membrane lipid composition of a Sulfurimonas species depend on the electron acceptor used for sulfur oxidation
Source: ISME Commun. 2022 Dec 24;2:121. doi: 10.1038/s43705-022-00207-3 (PMC9789136; doi:10.1038/s43705-022-00207-3)
Supplement: Supplementary file 1 — Supplementary Information [file 43705_2022_207_MOESM1_ESM.docx]

**Supplementary Information**


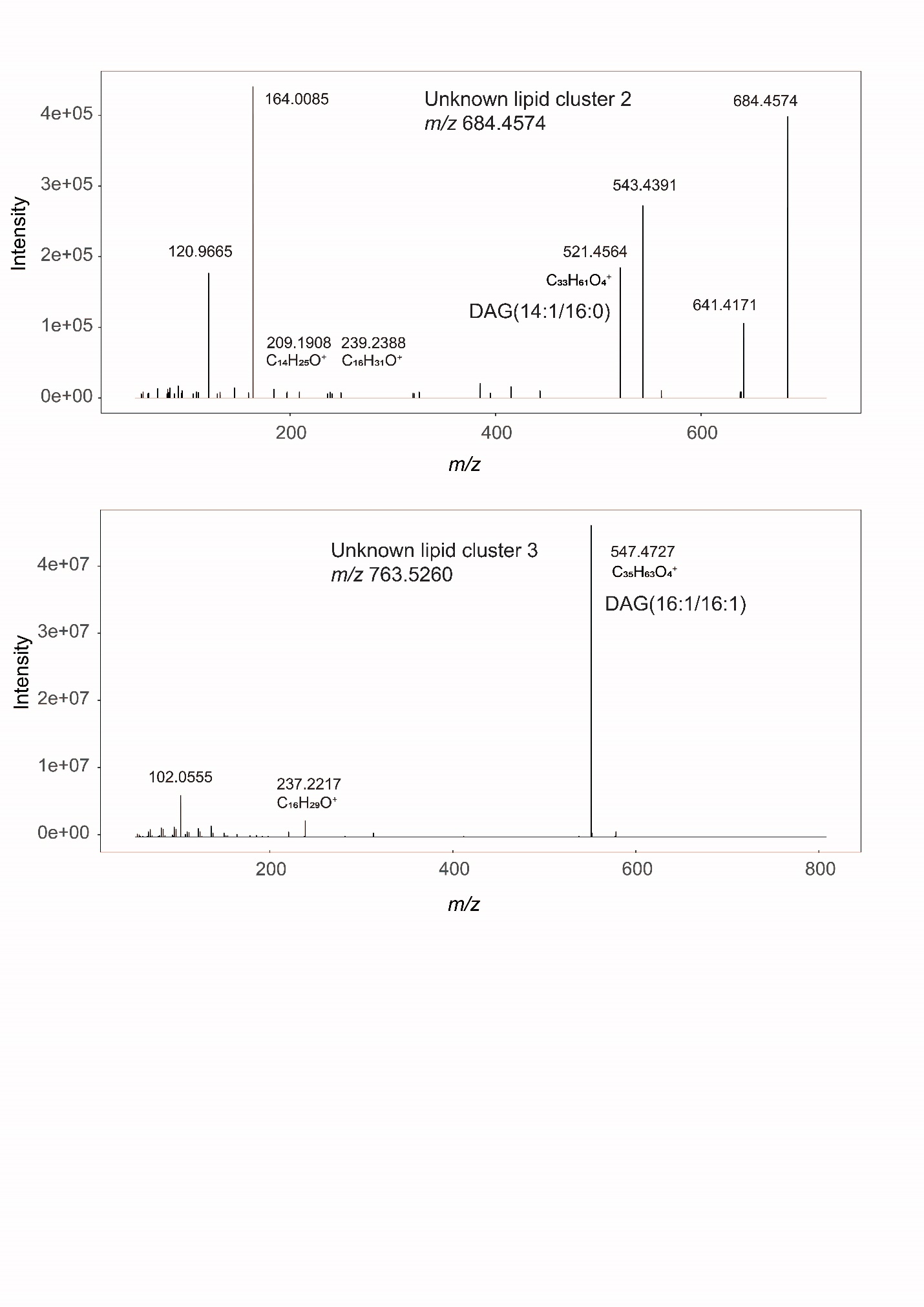


**Figure S1. MS^2^ spectra of representative unknown lipids in the subnetwork of unknown 2 and unknown 3 (Fig. 1A), respectively. Both of them have a diacylglycerol core lipid.**


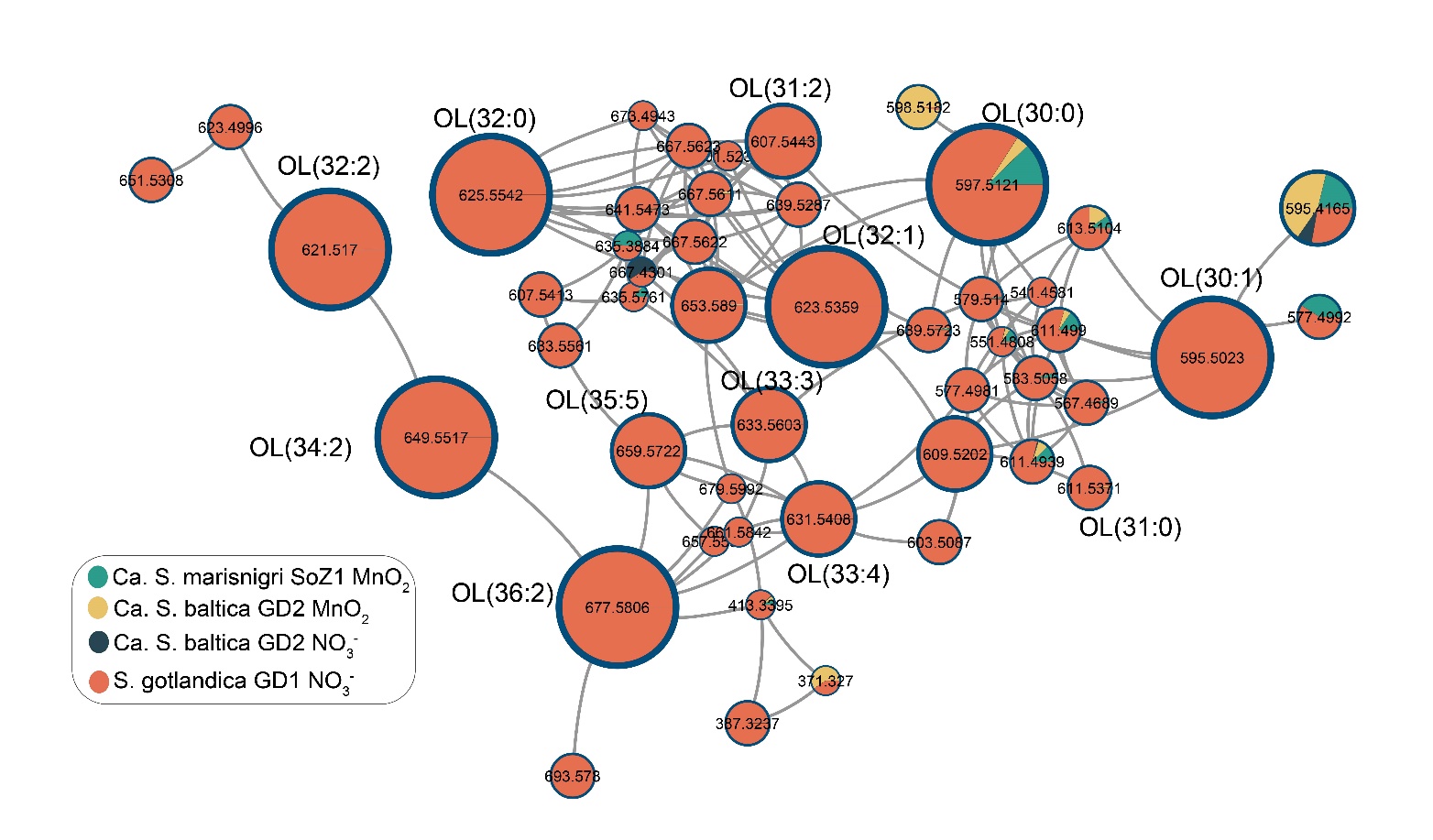


**Figure S2. Molecular subnetwork of ornithine lipids (OLs).** Pie charts shown in subnetwork are the same as nodes shown in Fig. 1A, representing lipid species. The size of the pie charts represents the summed intensity of all samples. Colors of pie chart represents the fractional abundance of this lipid species among all the *Sulfurimonas* species or different treatment.


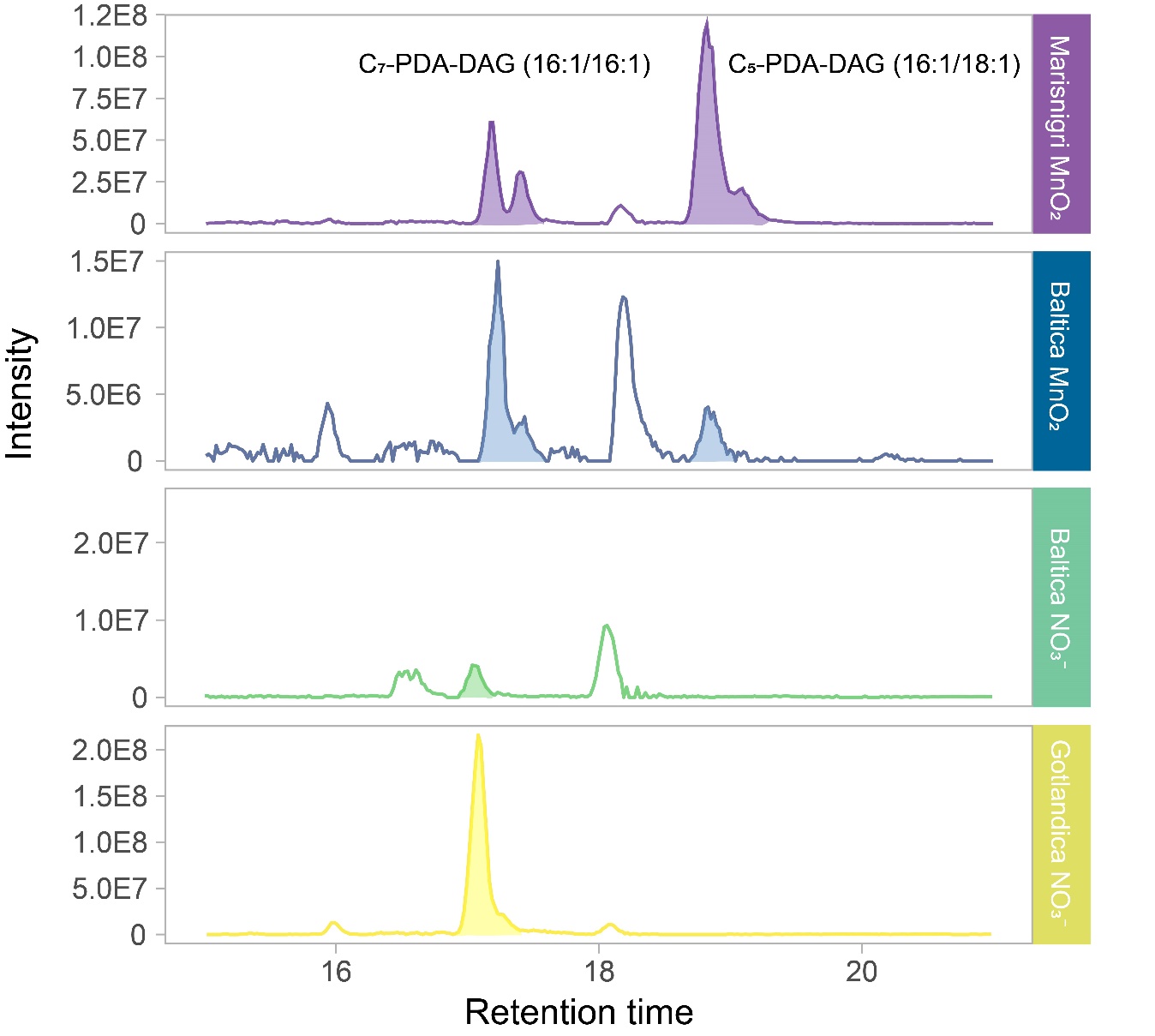


**Figure S3. Mass Chromatograms of two PDA-DAGs that have the same molecular mass (*m/z* 771.5647) but differ in the size of the alkyl chains of the polar headgroup and the number of carbon atoms of the acyl moieties.** *Ca.* S. baltica GD2 and *Ca.* S. marisnigi SoZ1 grown with $\mathrm{MnO}_{2}$ as electron acceptors can produce both of the PDA-DAGs (C_5_-PDA-DAG and C_7_-PDA-DAG), *Ca.* S. baltica GD2 and *S.* gotlandica GD1 grown with $\mathrm{NO}_{3}^{-}$ can only produce C_7_-PDA-DAG.


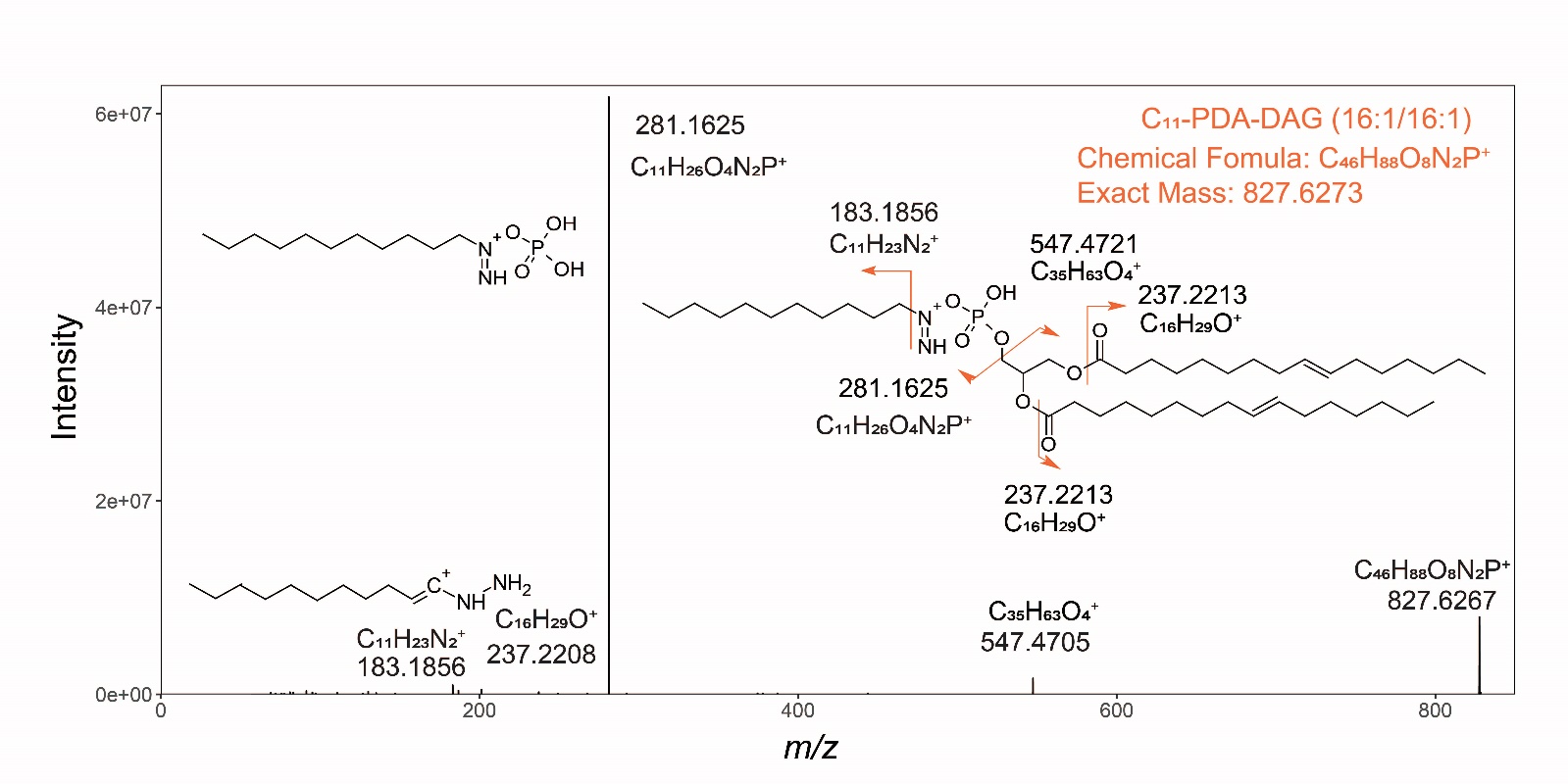


**Figure S4. MS^2^ spectra of C_11_-PDA-DAG (*m/z* 827.6273).**
